# Genetic parallelism between European flat oyster populations at the edge of their natural range

**DOI:** 10.1111/eva.13449

**Published:** 2022-08-06

**Authors:** Sylvie Lapègue, Céline Reisser, Estelle Harrang, Serge Heurtebise, Nicolas Bierne

**Affiliations:** ^1^ MARBEC, Univ Montpellier, CNRS Ifremer, IRD Montpellier France; ^2^ Ifremer, ASIM La Tremblade France; ^3^ ISEM, Univ Montpellier, CNRS, EPHE, IRD Montpellier France

**Keywords:** European range, genetic parallelism, natural populations, *Ostrea edulis*, restoration, SNPs

## Abstract

Although all marine ecosystems have experienced global‐scale losses, oyster reefs have shown the greatest. Therefore, substantial efforts have been dedicated to restoration of such ecosystems during the last two decades. In Europe, several pilot projects for the restoration of the native European flat oyster, *Ostrea edulis*, recently begun and recommendations to preserve genetic diversity and to conduct monitoring protocols have been made. In particular, an initial step is to test for genetic differentiation against homogeneity among the oyster populations potentially involved in such programs. Therefore, we conducted a new sampling of wild populations at the European scale and a new genetic analysis with 203 markers to (1) confirm and study in more detail the pattern of genetic differentiation between Atlantic and Mediterranean populations, (2) identify potential translocations that could be due to aquaculture practices and (3) investigate the populations at the fringe of the geographical range, since they seemed related despite their geographic distance. Such information should be useful to enlighten the choice of the animals to be translocated or reproduced in hatcheries for further restocking. After the confirmation of the general geographical pattern of genetic structure and the identification of one potential case of aquaculture transfer at a large scale, we were able to detect genomic islands of differentiation mainly in the form of two groups of linked markers, which could indicate the presence of polymorphic chromosomal rearrangements. Furthermore, we observed a tendency for these two islands and the most differentiated loci to show a parallel pattern of differentiation, grouping the North Sea populations with the Eastern Mediterranean and Black Sea populations, against geography. We discussed the hypothesis that this genetic parallelism could be the sign of a shared evolutionary history of the two groups of populations despite them being at the border of the distribution nowadays.

## 1 INTRODUCTION

Although all marine ecosystems have experienced global‐scale losses (e.g. Waycott et al., 2009 for seagrass; Wernberg et al., 2019 for kelp forests), oyster reefs have shown the greatest, estimated to 85% at a global scale. In many bays, more than 99% of oyster reefs have been lost and are functionally extinct (Beck et al., 2011). Beyond their commercial food value, oysters are ecosystem engineers, as they produce reef habitat for entire ecosystems and their ecological role could be compared with coral reefs in tropical regions. Therefore, substantial efforts have been dedicated to restore such ecosystems in the past two decades, but the accelerated footprint of climate change and increasing anthropogenic pressures on marine life raise an urgent need for more restoration programmes worldwide (Duarte et al., 2020). In such programmes, the focus is often placed on the population or the community, but rarely on the ecosystem (Rinkevich, 2021). Moreover, while a multispecies approach to restoration, as was performed for the Australian flat oyster, *Ostrea angasi*, has proven to accelerate recovery of extinguished oyster reefs (McAfee et al., 2021), restoration often remains focused on a single‐species.

**FIGURE 1:**
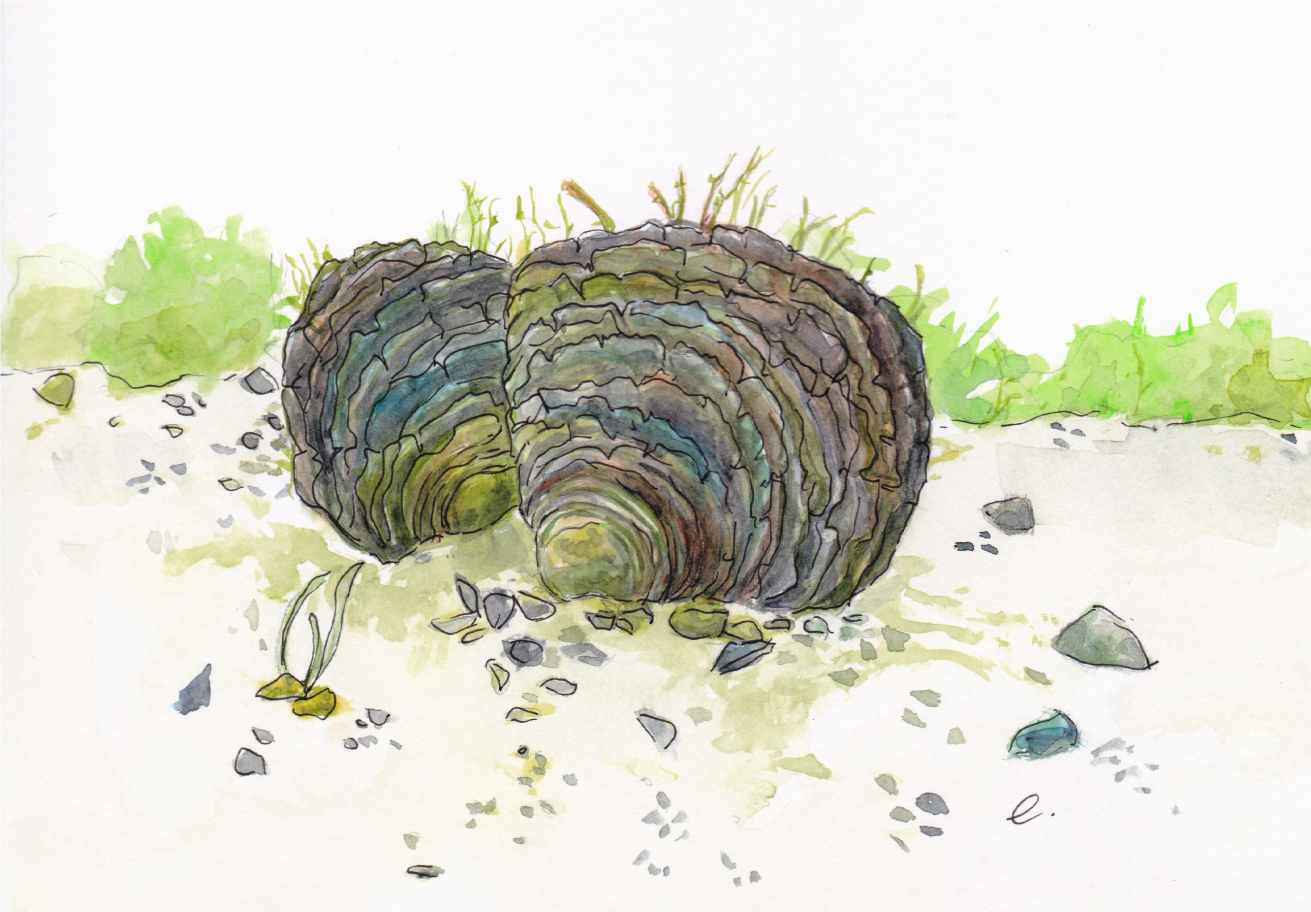
Illustration of two European flat oysters, *Ostrea edulis*. © Emma Rozis, Illumer.

In Europe, the native European flat oyster, *Ostrea edulis* (Figure 1), has been heavily fished as a valuable food resource over the last centuries, dating as far as the Mesolithic (some shells were identified in kitchen middens at least 6000 years ago; Gutiérrez‐Zugasti et al., 2011). By the end of the 19th century, its natural range was encompassing the North‐Eastern Atlantic (from Norway to Morocco) and the whole Mediterranean basin and Black Sea (Lapègue et al., 2006). From 1850 to 1920, overexploitation of natural oyster beds as well as bottom trawling for other target species led to their extinction in most European regions, which is also consistent with natural history collections in the North Sea (Hayer et al., 2019). In regions where a commercial production remained (based on managed oyster beds), two main diseases caused by parasites (principally *Bonamia ostreae*) resulted in high mortalities (Arzul et al., 2006) that increased the degradation of oyster beds or even led to their disappearance. *O. edulis* survived in coastal and estuarine areas mainly thanks to the management of oyster beds for commercial purposes, and the Oslo‐Paris (OSPAR) Convention for the protection of marine environment of the North‐East Atlantic included this species on the list of threatened and declining species and habitats. Although millions of oysters were regularly moved within and between countries over the last 200 years, such translocations mainly failed (Bromley et al., 2016). Several recent pilot projects have begun in UK, France, the Netherlands, and Germany and led to the creation of the Native Oyster Restoration Alliance (NORA, https://noraeurope.eu/) whose members made several recommendations for the settlement of a European flat oyster restoration program (Pogoda et al., 2019). They encompassed recommendations on both the sites (identify and create suitable sites for restoration, provide suitable substrate for successful recruitment, respect Bonamia‐free areas) and the animals (produce enough oysters, preserve genetic diversity), while recognizing the need to create and conduct common monitoring protocols. Shellfish restoration programs can be supported by mass release of hatchery‐produced juveniles, although genetic impacts of such practices are not fully understood and rarely monitored. In the United States, over the past century, fishery landings of the eastern oysters *Crassostrea virginica* have declined by >90% in most of the Atlantic coastal states' estuaries (MacKenzie, 1996), and >50% of the area of oyster reef habitat has disappeared from Chesapeake Bay (Rothschild et al., 1994). After a pioneer restoration program in 2012 in this bay, a recent reevaluation of the effects of hatchery propagation and initial characterization of diversity on restored versus wild eastern oyster reefs suggests that local wild broodstock maintained genetic diversity in a restored reef population compared with proximal wild populations (Hornick & Plough, 2019). Moreover, when considering management issues of restoration programs, an initial and unmissable step is to test for genetic differentiation against homogeneity among the oyster populations potentially involved in such programs as performed for the eastern oyster species in the Canadian Maritimes (Bernatchez et al., 2019).

Several types of molecular markers have been used to characterize the genetic structuring and diversity of the European flat oyster populations or stocks at very different scales. Five microsatellite markers confirmed the European origin of oysters naturalized on the eastern coasts of Canada after their introduction in the 1970s (Vercaemer et al., 2006). They also detected lower genetic diversity in hatchery stocks than in wild or naturalized populations in Europe and Canada (Lallias, Boudry, et al., 2010; Vercaemer et al., 2006), a high genetic differentiation between Europe and Canada, and signs of inbreeding in the hatchery stocks. The effective number of breeders can be low especially in hatchery stocks (Lallias, Boudry, et al., 2010) but also in wild conditions (Lallias, Taris, et al., 2010) supporting the hypothesis of sweepstake reproductive success in this species (Hedgecock & Pudovkin, 2011). This needs to be taken into consideration and monitored when planning restocking.

At the European level, genetic structuring and diversity of *O. edulis* was analysed with allozymes (Blanc et al., 1986; Jaziri, 1990; Saavedra et al., 1993, 1995), mitochondrial DNA (mtDNA) (Diaz‐Almela et al., 2004), and microsatellites (Beaumont et al., 2006; Lallias, Boudry, et al., 2010; Launey et al., 2002; Sobolewska & Beaumont, 2005; Vera et al., 2016). All the studies globally agree with a weak but significant genetic differentiation between Atlantic and Mediterranean populations, following an isolation by distance model (Diaz‐Almela et al., 2004; Launey et al., 2002; Saavedra et al., 1995). Moreover, a distinct cluster was identified in Northern Sea with additional microsatellite markers (Vera et al., 2016). If the analysis performed with the mtDNA 12S‐rRNA gene confirmed such a general pattern (Diaz‐Almela et al., 2004), it also highlighted that “the geographically extreme populations sampled in Norway and Black Sea appeared differentiated from the others, with the dominance of a third group of haplotypes”. Therefore, a new sampling at the European scale was performed and new analyses using SNPs developed by Lapègue et al. (2014) were performed, in order to (1) confirm and study in more detail the general pattern already observed at this scale, (2) identify potential translocations that could be due to aquaculture practices, and (3) investigate the genomic characteristics of the populations at the border of the geographical range. The results should be useful at the start of restoration programs to enlighten the choice of the animals to be translocated or reproduced in hatcheries for further restocking.

## 2 MATERIALS AND METHODS

### 2.1 Biological material

**FIGURE 2:**
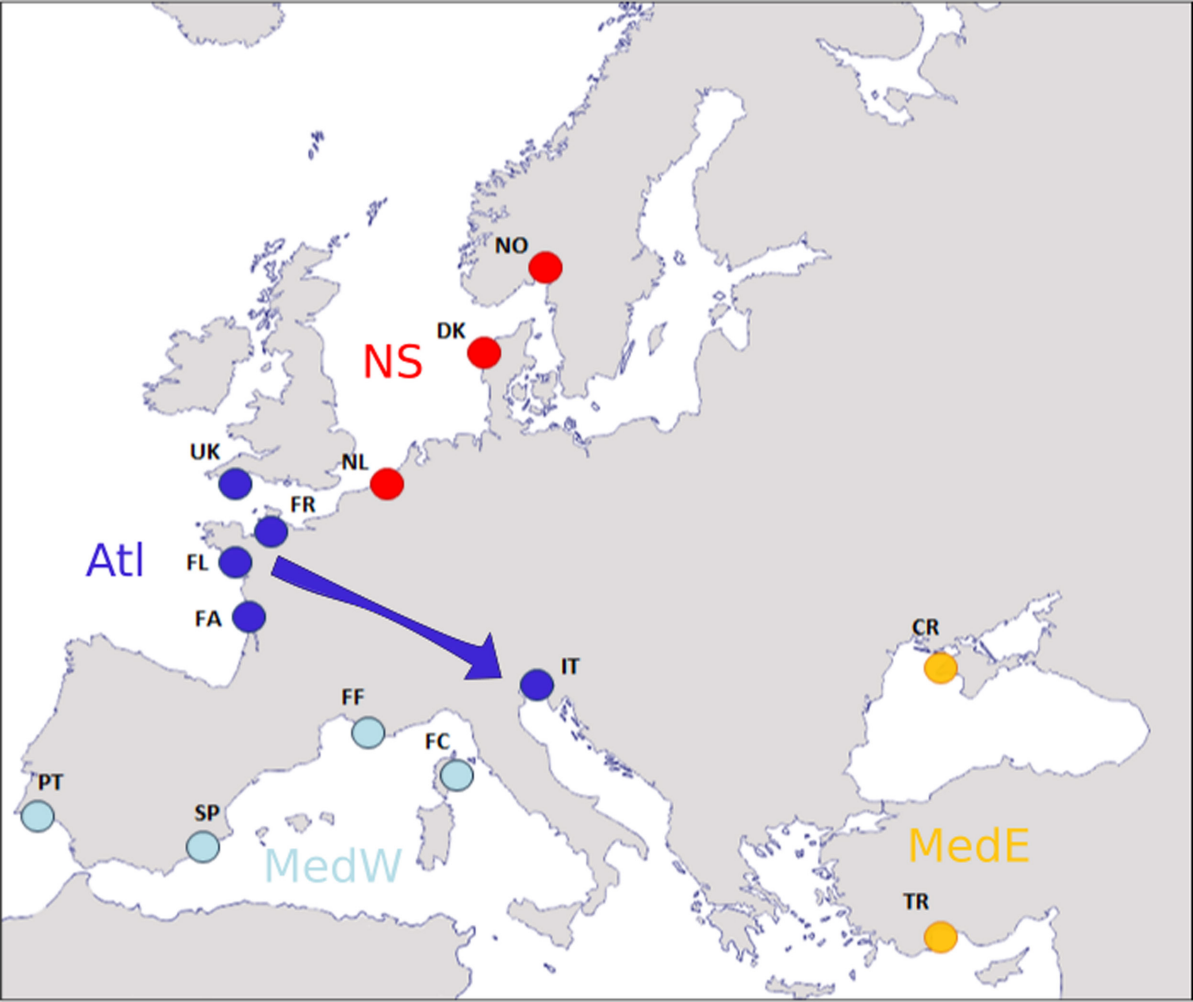
Locations of the flat oyster samples in Europe: In Norway (NO), Denmark (DK), Netherlands (NL), United Kingdom (UK), France (FR, FL, FA, FF, and FC), Portugal (PT), Spain (SP), Italy (IT), Turkey (TR), and Crimea (CR). The colour code corresponds to the four main clusters identified in the PCA: NS in red for North Sea, Atl in dark blue for Atlantic Ocean, MedW in light blue for the western part of the Mediterranean Sea, and MedE in orange, for the eastern part of Mediterranean Sea and Black Sea.

A total of 14 populations of *O. edulis* were sampled along European coasts, from Norway to Black Sea, 10 of them between 2005 and 2010. Four locations correspond to older samples (1996–2000) from Launey et al. (2002) (Figure 2, Table S1). The number of samples per population varied from 29 to 52 for a total number of 617 oysters.

### 2.2 SNP genotyping and functional annotation

For each individual, genomic DNA was extracted from 20 mg of gill tissue, mantle, or muscle using the QIAamp® DNA mini‐kit (Qiagen) according to the manufacturer’s recommendations. Quality and concentration were assessed on a 1% agarose gel and by using a NanoDrop Spectrophotometer ND‐2000 (Thermo Scientific). Concentrations were equilibrated to 100 ng/μl per DNA sample.

A set of 384 SNP markers was specifically developed on the European flat oyster (Lapègue et al., 2014), from direct sequencing (Harrang et al., 2013; Morga et al., 2012) and in silico analysis of RNAseq data (Gayral et al., 2013). In the first case, a total of 22 oysters (16 from natural populations and 6 belonging to the first generations of three selected families for resistance to bonamiosis) were used to investigate polymorphisms. In the second case, we investigated *O. edulis* transcriptome sequence data from eight individuals from the natural range. SNPs that were detected by sequencing a subset of individuals for candidate genes will be referred to as in vitro SNPs. Those that were detected by aligning different sequences from contig databases will be referred to as in silico SNPs. Fifty‐two in vitro SNPs were included in the array, representing 35 different gene fragments. A total of 1305 in silico SNPs fitted the proposed criteria for Illumina genotyping. Therefore, we chose the 332 in silico SNPs with the highest Illumina Functionality Score (>0.935) to complete the array.

The panel was genotyped with the Illumina GoldenGate technology (Illumina Inc.) according to the manufacturer’s recommendations, at the Genotoul platform (Genopole Toulouse Midi Pyrénées). The clustering was realized with the GenomeStudio software (Illumina Inc.). Reliability of SNP detection and coding were the same as what is reported in Lapègue et al. (2014). In particular, a quality score had been produced for each genotype. GenCall (quality metric that indicates the reliability of the genotypes called) and GenTrain scores (locus‐specific measure taking into account the quality and shape of the genotype clusters and their relative distances from one another) provide information on the reliability of SNP detection based on the distribution of genotypic classes (AA, AB, and BB). A GenCall score cutoff of 0.25 was applied to define valid genotypes for each SNP. Then only those which attained a minimum GenTrain score of 0.25 were kept. These scores are the same stringent thresholds as those applied in previous studies on other species (i.e. in pine: Lepoittevin et al., 2010).

We used the genome assembly of *Crassostrea gigas* (cgigas_uk_roslin_v1, Genbank accession code GCF_902806645.1) in an attempt to map the loci corresponding to our 203 SNPs. The GFF file of the genome was used to functionally annotate those loci.

### 2.3 Linkage disequilibrium

We obtained the estimation of linkage disequilibrium (LD) through a correlation‐based test for loci with multiple alleles using the MCLD program available at https://brcwebportal.cos.ncsu.edu/zaykin/rxc (Zaykin et al., 2008). The distribution of pairwise *r* ^2^ (equivalent to the Pearson correlation coefficient) was visualized as a raincloud plot using the R code available at https://github.com/RainCloudPlots/RainCloudPlots/tree/master/tutorial_R.

The resulting pairwise *r* ^2^ value obtained were then submitted to the Tuckey’s fence method for outlier detection. This method considers as outlier a value that is higher than Q3 + *k*(IQR), where IQR = Q3–Q1 (Q1 is the first quartile, Q3 the third quartile, IQR the interquartile range, and *k*, a constant). Here, we set *k* = 3, to obtain only the strong outlier values. Loci showing *r* ^2^ value being outlier in at least two pairwise comparisons were retained. Pairwise *r* ^2^ values were then ordered according to the position of loci on the genetic map of *O. edulis* (Harrang et al., 2015) and plotted as a heatmap using the R package ggplot2.

### 2.4 Genetic diversity and structure

Genetix (4.05.2; Belkhir et al., 1996–2004) was used to report overall observed (*Hobs*) and expected (*Hnb*) heterozygosity for each population. Within‐ and between‐population components of genetic diversity were decomposed using a principal component analysis (PCA), using dudi.pca function of the R package Ade4.

Bayesian clustering of the genetic data was performed using Structure version 2.1 (Pritchard et al., 2000). We ran Structure with *K* (the assumed number of populations or genetic groups) varying from 1 to 10, with 10 runs for each *K* value, to find the *K* value with the highest posterior probabilities and used the Δ*K* statistics to evaluate the change in likelihood (Evanno et al., 2005) implemented in Structure harvester (Earl & vonHoldt, 2012). Our parameters were 10,000 burn‐in periods and 5000 Markov chain Monte Carlo repetitions after burn‐in. For the most likely number of clusters, we calculated the average result over 10 runs to get the final admixture analysis. The inferred ancestry of each individual to the *K* clusters was plotted with R package ggplot2.

An Analysis of MOlecular VAriance (AMOVA) was performed following Excoffier et al. (1992) with the function poppr.amova (with the ade4 implementation) of the R package poppr, based on the clusters defined by the Bayesian clustering. This analysis tests the differences among populations and/or groups of populations in a way similar to ANOVA, including evolutionary distances among alleles.

Evolutionary history of the genetic clusters was then investigated under a model of divergence and admixture events using the population graph approach implemented in Treemix (Pickrell & Pritchard, 2012). Default parameters were used, and we performed the analysis using a value of *m* (number of migration edges) ranging from 0 to 3.

We computed Patterson et al.’s f4 statistics (Patterson et al., 2012) as f4 = (p_NS ‐ p_Atl)(p_MedE ‐ p_MedW) with p_NS, p_Atl, p_MedE, and p_MedW being the allele frequency in the North Sea, Atlantic, Eastern, and Western Mediterranean Sea genetic clusters, respectively. As defined, f4 provides positive values when the differentiation is parallel, and negative values when the differentiation is antiparallel. The expectation in absence of coancestry correlation in a pure drift model is as many positive as negative values. An excess of positive values suggests shared ancestry caused by admixture or a shared history. In order to identify loci with outlier f4 values, we again applied the Tuckey's fence method with *k* = 3. Besides, thanks to the Fst function of the R package pegas, we computed, for each locus, the *F*_ST_ statistic, respectively, between the North Sea and Atlantic clusters, and Western and Eastern Mediterranean Sea clusters, in order to produce a *F*_ST_‐*F*_ST_ coplot as in Fraïsse et al. (2014).

### 2.5 Outlier loci

We conducted *F*_ST_ outlier tests mainly to illustrate that the observed *F*_ST_ variance is larger than a standard neutral model and to show that the most differentiated loci turn positives with such tests. We used two methods. First, we used BAYESCAN (Foll & Gaggiotti, 2008), a Bayesian method that uses a logistic regression model to estimate the posterior probability that a given locus is under selection. BAYESCAN was used with a burn‐in: 50,000; thinning interval: 10; sample size: 50,000; resulting total number of iterations: 550,000; nb of pilot runs: 20; length of each pilot run: 5000. We analysed the whole dataset as well as pairs of populations. The latter approach is sometimes used to avoid false positives produced by correlation in coancestry in multipopulations tests (Vitalis et al., 2001). Second, we used PCAdapt (Luu et al., 2017) using the most conservative method (“Bonferroni”) and alpha = 0.01. This method is a standard nonparametric outlier test very similar to Tuckey's method. We obtained outliers identified in pairwise comparisons with BAYESCAN and those that were identified by both tests using the whole dataset.

## 3 RESULTS

### 3.1 Genotypes

A total of 250 SNP markers (65.1%) gave reliable data. Among them, 203 SNPs (52.9%) were polymorphic and used for further analyses. The high rate of genotyping failure falls within the range of values seen in other nonmodel species but are very different from those obtained in model species (see discussion in Lapègue et al., 2014). Hence in nonmodel species, particularly, genotyping failures may be due to (i) low quality of SNP flanking sequences; (ii) the presence of an exon–intron junction near the SNP of interest potentially not detected in the case of transcriptomic data; or (iii) the assembly and/or amplification of paralogous sequences, leading to false‐positive SNPs. Moreover the final relatively low level of polymorphic success might be due to the fact that a low number of animals were used to develop the SNPs of the panel. However, the 250 SNPs kept were chosen based on a stringent GenTrain score completed by a careful visual inspection of the clusters in order to avoid genotyping biases. A total of 617 individuals, representing 14 different populations, were genotyped with these 203 SNPs.

### 3.2 Linkage disequilibrium

**FIGURE 3:**
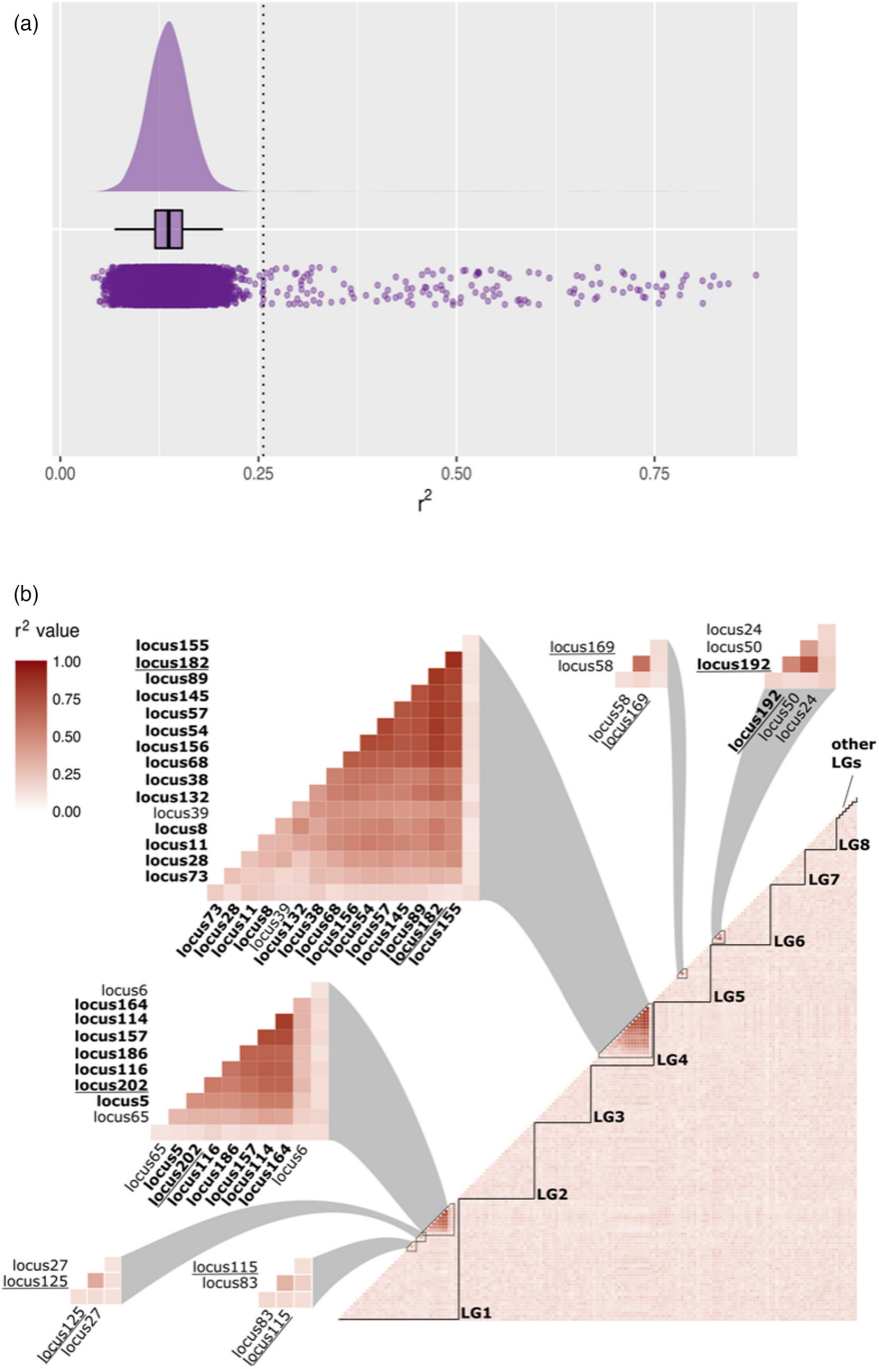
LD estimates through pairwise *r* ^2^ between markers. (a) Raincloud plot representation of the *r* ^2^ values with the dotted line indicating the threshold (*k* = 3) for the detection of outlier *r* ^2^ values. (b) Heatmap of the *r* ^2^ values with markers ordered according to the genetic map from Harrang et al. (2015). The 33 SNP loci detected with high LD between pairs of markers are grouped in 6 LD‐groups that are highlighted above the heatmap. The loci underlined are the representative of their LD‐group and the loci in bold are moreover outlier loci.

We detected 33 SNPs exhibiting high LD (*r* ^2^ values) between pairs of markers (Figure 3a). They split into six LD‐groups (Figure 3b) labelled by the name of one of the SNP of the group underlined. The biggest group named LDG_182 encompasses 15 markers, the second one named LDG_202 encompasses nine markers. Then the group named LDG_192 encompasses three markers and the three smallest groups encompasses, respectively, named LDG_115, LDG_125, and LDG_169 encompass two markers. SNPs of the two main LD‐groups (LDG_182 and LDG_202) were, respectively, mapped to linkage groups 4 and 1 on the genetic map of Harrang et al. (2015) suggesting that the high LD observed is due to physical linkage on the chromosome (Figure 3b). We used the genotype at one SNP of each of the two biggest LD‐groups (LDG_182 and LDG_202) to visualize the effect on a PCA (Figure S1). As expected, we observed that LD‐groups have a strong influence on the PCA. Three groups of individuals were observed on the plan defined by PC1 and PC3 with little effect of geographic sampling (Figure S1a). Once coloured according to their genotypes these groups of individuals proved to correspond to the three genotypes at LDG_182, while LDG_202 structured point clouds in an orthogonal manner (Figure S1b). Finally, SNPs of the same LD‐group displayed a common pattern of allelic frequencies within populations: NS (North Sea) and MedE (eastern part of Mediterranean Sea and Black Sea) populations showed a high frequency for one allele (Figures S2 and S3). For LDG_202, we observed more specifically that NO population (from North Sea) and MedE populations showed a high frequency for one allele.

We successfully managed to locate 50 out of our 203 loci on the *C. gigas* genome (Table S2). Interestingly, SNPs found in LD in *O. edulis* (Figure 3b) were also located on the same linkage group on *C. gigas* genome (LDG_182 on LG4 for *O. edulis* and LG4 for *C. gigas*, LDG_202 on LG1 for *O. edulis* and LG10 for *C. gigas*), showing that synteny was likely conserved for those chromosomal regions between the two species.

In order to avoid any bias due to the large number of linked loci, only one marker of each of the six LD‐groups (the representatives of the LD‐groups presented above as underlined in Figure 3b) was kept for the genetic structuring statistics, leaving a dataset of 176 SNP markers.

### 3.3 Genetic diversity and structure

*Hnb* and *Hobs* varied from 0.311 to 0.393 and 0.308 to 0.383, respectively (Table S3). The lowest values were observed in eastern Mediterranean Sea (TR) and Black Sea (CR) populations and then in a less important way in Norway (NO). Note that SNPs were ascertained without samples from the eastern Mediterranean Sea and Black Sea and given these populations are well differentiated from the others (see below), the lower heterozygosity found in these populations could be due to a slight ascertainment bias.

**FIGURE 4:**
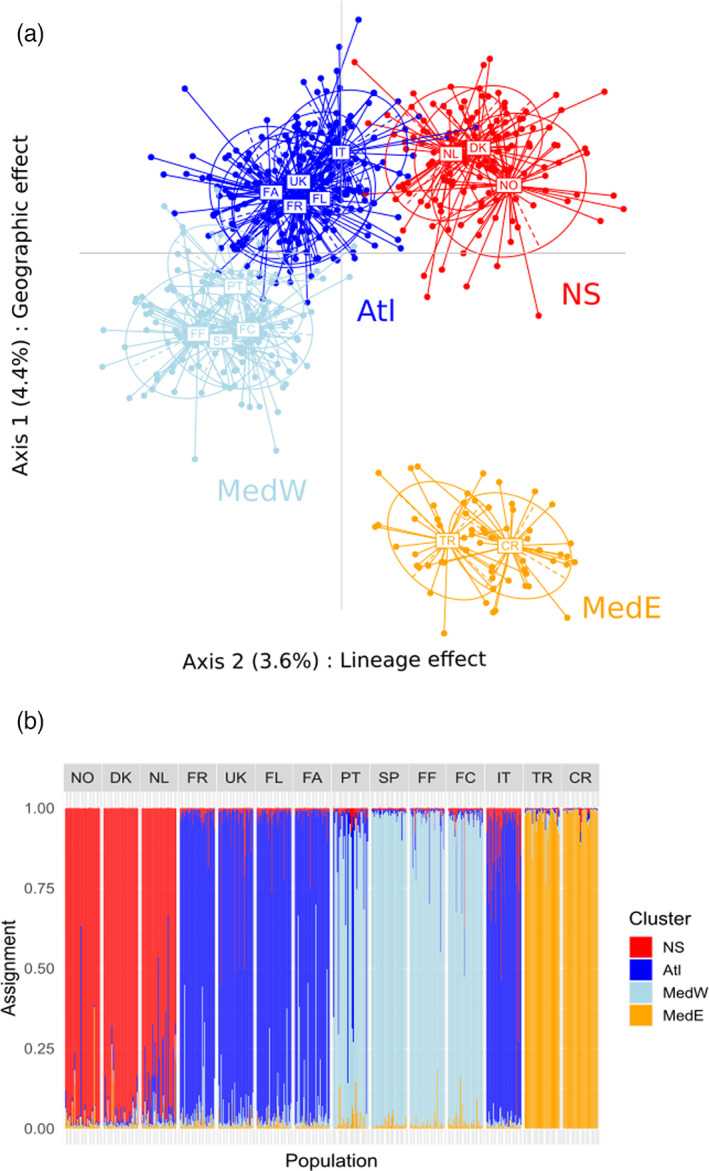
Genetic structure representations for the 14 populations and 176 SNP markers. (a) Two first axes of the PCA showing four main clusters. (b) Structure analysis indicating the probability of assignment to same four clusters. The four clusters are named NS for North Sea, Atl for Atlantic Ocean, MedW for the western part of the Mediterranean Sea, and MedE, for the eastern part of Mediterranean Sea and Black Sea.

The PCA and Structure analyses performed on the dataset with 176 markers gave the same global result of four main clusters named NS for North Sea, Atl for Atlantic Ocean, MedW for the western part of the Mediterranean Sea, and MedE, for the eastern part of Mediterranean Sea and Black Sea (Figure 4). The four clusters mainly encompassed populations from a same geographical area: NO, DK, and NL populations in the NS cluster, FR, UK, FL, FA for the Atl cluster, SP, FF, and FC for the MedW cluster, and TR and CR for the MedE cluster. It must be noted that the Italian population (IT), located in the Adriatic Sea, was grouped with the Atlantic populations and subsequently coloured in dark blue in Figure 4 in order to emphasize its genetic similarity with Atlantic samples. In the same way, the Portuguese population (PT), located on the Atlantic coast, clustered with the western Mediterranean populations and was subsequently coloured in light blue in Figure 4. PCA analysis showed that the two first axes mainly explained the genetic variance observed and 8% of the total variance. On axis 1 (explaining 4.4%), the populations followed a geographic distribution from the Black Sea and eastern Mediterranean Sea (MedE cluster) on the lower part of the graph to the North Sea populations (NS cluster) on the upper part of the graph. On axis 2 (explaining 3.6%), the two clusters at the extreme parts of the distribution of the species (MedE and NS) appeared on the right, whereas the two clusters encompassing populations in the internal part of the distribution (MedW and Atl) appeared on the left.

The AMOVA on the four previously identified clusters (excluding IT and PT samples) also gave the same result with 8.7% of the variance explained between clusters and only a small part of the variance (1.8%), still significantly different from zero, explained between populations of the clusters (Table S4). However, considering this low level of variation between populations within a same cluster, we decided to apply the TREEMIX analysis to the four clusters but excluding PT and IT populations.

**FIGURE 5:**
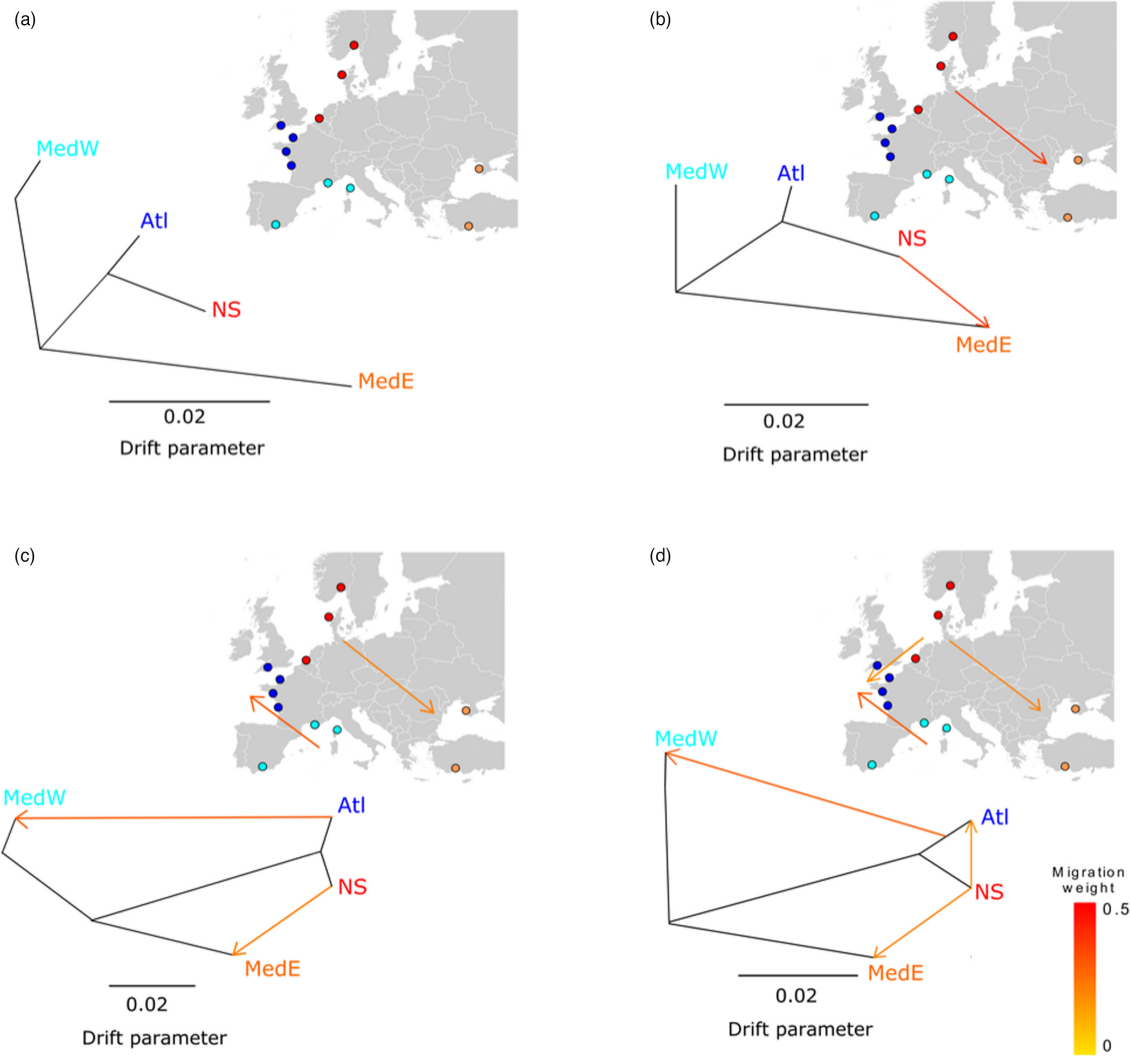
Evolutionary history of the genetic clusters using the population graph approach of TREEMIX. Population trees, respectively, inferred without accounting for migration (a), with 1 (b), 2 (c), or 3 (d) events of migration. After an initial clustering according to geography (a), two events of migration were identified first between the two peripheral populations, NS and the MedE, revealing genetic similarity despite their geographic distance (b), and the second event between Atl and MedW populations (c). A third migration event was identified between clusters that are in contact, NS and Atl (d).

The population tree inferred using TREEMIX without accounting for migration (Figure 5a) is consistent with the analyses performed with the PCA and Structure analyses, with populations clustering according to geography. Atlantic Ocean and North Sea clusters are branching together as observed on the first axis of the PCA. However, when considering models with migration, events of migration were identified against geography but in accordance with the grouping observed in PCA axis 2. Two events of migrations were identified within clusters defined by PCA axis 2, the first between the two peripheral populations, NS and MedE, revealing genetic similarity despite their geographic distance and the second event between Atl and MedW populations (Figure 5b,c). A third migration event was identified between clusters that are in contact, NS and Atl (Figure 5d).

### 3.4 Outlier detection and functional annotation

A total of 28 SNPs was identified by both BAYESCAN and PCAdapt and 17 of those SNPs were identified in pairwise comparisons (8 between NS and Atl and 9 between MedE and MedW). If we conservatively focus on the 28 SNPs identified with the two methods, they encompassed 14 of the 15 SNPs of LDG_182 (all but SNP39), 7 of the 9 SNPs of LDG_202 (all but SNP6 and SNP65), and only SNP192 of LDG_192. Those markers were indicated in bold on Figure 3b. Furthermore six other outliers were detected: SNP33, 64, 70, 94, 95, and 176. The 21 SNP outliers from the two major groups of linked markers, LDG_182 and LDG_202, showed the same global pattern of allelic frequencies with a higher frequency in NS and MedE clusters and a lower frequency in Atl and MedW clusters (Figures S2 and S3).

Of the 28 SNPs, 13 were successfully mapped against *C. gigas* genome. Functional annotation of these 13 SNPs was performed using the GFF file given alongside the genome. These SNPs are mostly located in exonic sequences of genes involved in pre‐ and posttranscriptional control and modification of RNA products, homeostasis and ER stress alleviation, and respiration (Table S5).

### 3.5 Genetic parallelism

**FIGURE 6:**
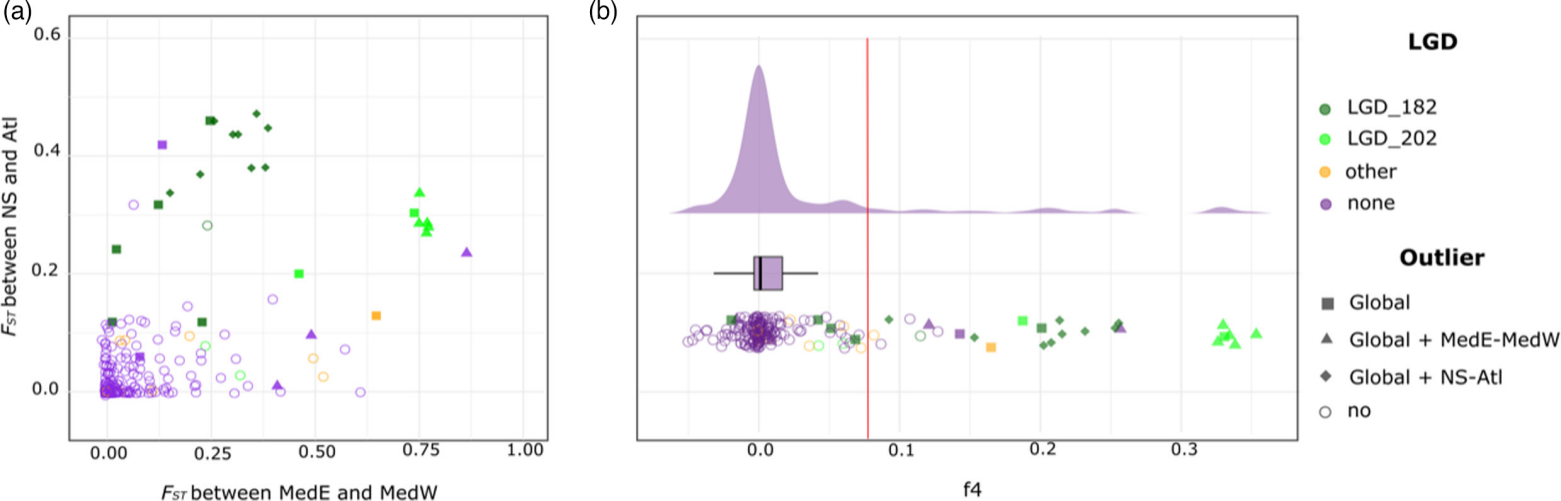
Genetic parallelism revealed by the plotting of the *F*_ST_ *‐F*_ST_ coplot (a), and the f4 distribution (b). Each shape is a SNP marker. The colours indicate the belonging of the SNP to an LDG or not. The filled shapes correspond to outlier SNPs in the global and pairwise analyses. The red vertical line in the f4 distribution indicates the threshold (*k* = 3) for the detection of outlier *r* ^2^ values.

Figure 6 shows *F*_ST_ *‐F*_ST_ coplot (Figure 6a) of the 203 markers (Fraïsse et al., 2014; Riquet et al., 2019) and the distribution of f4. These two graphs show a tendency for genetic parallelism, especially for the most differentiated loci, that are also *F*_ST_ outliers and belong to the two main groups of linked markers (LDG_182 and LDG_202). This illustrates the lineage effect of PCA axis 2. Hence populations at the edge of the distribution in the North Sea and the Eastern Mediterranean Sea have similar allele frequencies, against geography (Figures S2 and S3), for a subset of loci that also tends to be the more differentiated. Note that a few outliers were observed that do not follow this pattern of parallel differentiation between clusters but explain the local differentiation of one genetic cluster against the other three.

Interestingly, when we removed outlier loci in the PCA analysis, we still observed the separation of clusters on PCA axis 2 (Figure S4). In this PCA made with 170 non outlier markers, the first axis is still explaining 4.1% of the variance (instead of 4.4%), and the second axis 3% (instead of 3.6%), but the global pattern is conserved. This can also be observed on Figure 6b where purple circles, that correspond to nonlinked nonoutlier loci, show a distribution slightly biased towards positive f4 values, although the pattern is mainly visible with stronger levels of differentiation.

## 4 DISCUSSION

As is often the case for marine species (El Ayari et al., 2019; Gagnaire et al., 2015), the European flat oyster is subdivided by genetic barriers into a mosaic of near‐panmictic genetic clusters. We can define four clusters, two on the Western Coasts of Europe and two in the Mediterranean Sea. These four clusters can be considered as Evolutionary Significant Units (ESUs) and should accordingly be managed with dedicated conservation strategies. Interestingly, different parts of the genome revealed contrasted patterns of genetic structure. Most SNPs revealed low divergence between the four clusters and a structure that mainly follows geography. Conversely, groups of SNPs in strong LD and closely linked together on a genetic map revealed (i) outlying levels of genetic differentiation and (ii) patterns of genetic parallelism tending to group the two clusters at the extremity of the geographic range (North Sea and Eastern Mediterranean Sea).

### 4.1 Confirmation of a geographical pattern

Despite a supposedly high gene flow due to pelagic larval dispersal, *O. edulis* is not constituted by a single panmictic unit (Launey et al., 2002). Our study identified four different clusters ordered along a continuum ranging from the North Sea to the Black Sea, through the Atlantic Ocean, Western Mediterranean Sea, and Eastern Mediterranean Sea. Based on a close geographic sampling design (with however more recent samples except in Norway, Spain, and Crimea), our results partly agree with Launey et al. (2002) who identified three groups of populations with five microsatellite markers: Atlantic populations, Western Mediterranean populations and Eastern and Black sea populations. Thanks to a higher number of nuclear markers used in our study (203 SNPs), North Sea and Atlantic populations appeared differentiated, as also observed by Vera et al. (2016) with 16 microsatellite markers. However, the different sampling strategy between Vera et al. (2016) and our study did not allow to totally compare the results. Indeed, while Vera et al. (2016) identified a cluster in Spain, we did not sample in Galicia. However, we identified two clusters in western Mediterranean Sea on one side, and Eastern Mediterranean and Black Seas on another side, while Vera et al. (2016) did not sample in Mediterranean nor Black Sea. Furthermore, the heterozygosity estimates agree with this geographic pattern. As the SNPs were developed mainly on Atlantic and Western Mediterranean populations, highly differentiated with the Eastern Mediterranean and Black Sea populations, *Hnb* is expected to be lower in those later populations and in a lesser extend in the North Sea populations, which is currently observed.

An important discrepancy was observed for the Italian sample in the Venice Lagoon. While this area has been grouped with the Mediterranean populations in Launey et al. (2002), it clearly appears clustered with Atlantic populations in our study. However, the oysters were not sampled the same year and potentially not exactly at the same place. This discrepancy could be explained by the consequences of human activities, particularly the translocation of animals for aquaculture purposes (Johannesson et al., 1989; Saavedra et al., 1995; Šegvić‐Bubić et al., 2020). Indeed, even if official data concerning shellfish translocation are not totally reliable and sometimes underestimated (Bromley et al., 2016), Paquotte (1986) reported the occurrence of cupped oyster (*C. gigas*) translocation between France and Italy, particularly in the lagoon of Venice. Consequently, it would be possible that European flat oysters translocated at the same time as cupped oysters. This example illustrates the difficulty to disentangle long‐term evolutionary history and human activities as factors influencing the population genetic structure of species of interest for aquaculture (MacKenzie et al., 1997).

Globally, about 9% of the genetic variance is observed between the four clusters and only 2% between populations within clusters. Those clusters are distributed over extended regions. Unfortunately, the genetic discontinuities are difficult to delineate finely based on our sampling design. Some may however correspond to usual delimitations between vicariant marine clusters such as North Sea and Atlantic Ocean, Atlantic Ocean and Mediterranean Sea, and between Mediterranean and Black seas (Riquet et al., 2019; Woodall et al., 2015).

The limit between the North Sea and Atlantic clusters is likely to take place in the English Channel, maybe at the straights of Dover which coincide with a biogeographic boundary between the English Channel and the North Sea. Such a pattern has already been identified in several species, such as the common goby (*Pomatoschistus microps*, Gysels et al., 2004) or the common sole (*Solea solea*, Diopere et al., 2018) but other authors localized a genetic break within the English Channel, between French coasts of Normandy and Southeast Brittany in other species (*Mytilus* sp., Simon et al., 2021; *Pectinaria koreni*, Jolly et al., 2005). In the European flat oyster, the different samplings do not allow for the identification of the geographical location nor the extent of the “border” between the two clusters. However, considering the different projects of restoration planned or already undergoing (https://noraeurope.eu/restoration‐projects/projects‐overview/), it might be of importance to further investigate the genetic status of the populations in this area.

A biogeographic boundary has previously been mentioned in *O. edulis* at the Strait of Gibraltar (Saavedra et al., 1993, 1995). It was tested and identified in a brittle star species (*Ophioderma longicauda*, Boissin et al., 2011). However, in most of the studies in the Atlantic‐Mediterranean region, the Almeria‐Oran Front is the most likely cause of the observed structure (review in Patarnello et al., 2007). In our study, the genetic similarity between populations of Portugal, Spain, Fos‐sur‐Mer and Corse in France, suggests that the Strait of Gibraltar is not involved in the genetic structure of *O. edulis*. Furthermore, as also observed by Launey et al. (2002), the Portuguese sample (PT) is more related to the Western Mediterranean populations than the Atlantic ones (Figure 4a), and in Figure 4b, we can observe a signal of admixture indicating gene flow between the two clusters in this area. Besides, Vera et al. (2016) identified a Spanish cluster along the Galician coast. Taking those two observations into account, a more specific study around the Iberian Peninsula would be of interest to better characterize the genetic structure of natural populations of this area.

Finally, a major discontinuity was observed between populations of the Western Mediterranean Sea and populations of the Eastern Mediterranean Sea (Turkey) and Black Sea (Crimea). In the literature, two potential biogeographical barriers are discussed. The most western is located at the Siculo‐Tunisian Strait and is particularly involved in population structure of the sea bass (*Dicentrarchus labrax*, Bahri‐Sfar et al., 2000), cockle (*Cerastoderma glaucum*, Tarnowska et al., 2010) and clam (*Ruditapes decussatus*, Gharbi et al., 2011). The second is located at the Front of the Aegean Sea and the Cyrenaica Sea (Peloponnese break) in the Ponto‐Caspian region and was previously discussed in the cockle (Nikula & Väinölä, 2003; Tarnowska et al., 2010), the brittle star (*Ophioderma longicauda*, Boissin et al., 2011), and the damselfish (*Chromis chromis*, Domingues et al., 2005). In our study, it is not possible to state where the genetic discontinuity is precisely located. Here again, specific samplings need to be undertaken to better characterize the position of the genetic break.

Several hypotheses could explain the geographical patterns detected in our study. As mentioned above, the discontinuities between clusters revealed by a spatial differentiation matches with already described oceanographic barriers. The coupling hypothesis (Bierne et al., 2011) proposes that, although the boundaries between clusters could be due to exogenous barriers (oceanic fronts, temperature, salinity, presence of pathogens,…), “barrier loci” that could restrict gene flow on a large part of the genome (endogenous factors) could also, and mainly, contribute to the barrier to gene flow (Gagnaire et al., 2015; Le Moan et al., 2016; Ravinet et al., 2017; Rougemont et al., 2017). Here, we do observe a large number of markers that show restricted recombination (strong LD) across most of LG4, and almost half of LG1 on the genetic map of *O. edulis*. These regions might contain such “barrier loci”.

### 4.2 Parallel evolution at the edges of the distribution area

If about half of the variance is explained by the geographic pattern, a second half is due to parallel evolution of populations at the edges of the distribution area, that is to say populations of the North Sea (NS) and Eastern Mediterranean Sea and Black Sea (MedE) (lineage effect in Figure 3a). More than 10% of markers (28 among 203 SNPs) show comparable allelic frequencies between NS and MedE populations. While the other SNPs tend to group populations according to geographic distance, those 28 SNPs group together populations from NS and MedE, the most distant geographically and theoretically the least likely to exchange genes. *O. edulis* thus seems to show a discrepancy in evolutionary histories depending on the genomic regions considered in our dataset. This observation is in accordance with the results obtained, respectively, by Launey et al. (2002) and Diaz‐Almela et al. (2004). Such patterns have already been observed between the North Sea and Eastern Mediterranean Sea in the long‐snouted seahorse (Riquet et al., 2019), the harbor porpoise (*Phocoena phocoena*, Fontaine, 2016), or the European lobster (*Homarus gammarus*, Jenkins et al., 2019). More generally, many studies have demonstrated contradictory evolutionary histories depending on the genomic regions considered (Harrison & Larson, 2016; Martin et al., 2013), sometimes grouping geographically distant populations according to specific ecosystems such as between freshwater, lagoon, or coastal habitats and marine habitats (Jones et al., 2012; Le Moan et al., 2016; Riquet et al., 2019; Rougemont et al., 2017).

Two main hypotheses can be put forward to explain the discordant evolutionary histories observed within the *O. edulis* genome. First, independent convergent selection of the same alleles in the two border populations could explain this situation. According to this hypothesis, individuals from populations of NS and MedE could have been subjected to comparable selection pressures responsible for the increase in frequency of alleles located in the same chromosomal regions by direct selection and genetic hitchhiking of flanking regions. Selection would then have acted either on pre‐existing genetic variation or on alleles shared due to gene flow (“standing genetic variation or flowing genetic variation”, Welch & Jiggins, 2014). Adaptation of these populations to a similar environment would therefore have led to convergence at selected loci, and this despite the apparent difference in environments encountered by *O. edulis* in NS and in MedE populations. Functional annotation of the outlier SNPs grouping NS and MedE populations shows that SNPs mostly fall in exonic regions of genes which might be due to the fact that most of the SNPs of the panel were designed from transcriptomic data. However, it does not specifically give answers to the role of those genes in a putative local adaptation to the environment of both populations. Further studies centered on those genomic regions using phenotyping and genome scans will be necessary in all populations. In addition, given the size of the putative inversions, too many genes are sitting there for a functional annotation to help reveal which of those genes are targeted by selection.

The second hypothesis proposes that this parallel pattern of genetic differentiation is the genetic legacy of a shared evolutionary history between the two lineages at the border of the distribution range. According to this hypothesis the clusters of NS and MedE would derive from an ancestral lineage and would therefore share genetic similarities in genomic regions that kept the signal. The geographic proximity of the NS lineage with the Atlantic lineage (Atl) and that of the MedE lineage with the West Mediterranean lineage (MedW) would be explained by secondary gene flow that has gradually eroded the ancestral differentiation. SNPs showing genetic parallelism between border populations would have resisted secondary intergradation and retained the genetic proximity of these clusters (see Bierne et al., 2013). Those scenarios are difficult to discriminate as they can converge towards a similar pattern, and they are not mutually exclusive (adaptive introgression could have occurred during an ancient contact between the NS and the MedE lineages). A more in‐depth sequencing of the genome together with appropriate demographic reconstruction will be needed to settle the question.

Although we used only 203 SNPs in our study, we were able to demonstrate that outlier SNPs often cluster in genomic islands of differentiation (Figure 5). We identified two groups of linked markers, respectively, composed of 15 and 9 SNPs that (i) delineate groups of genotypes in the PCA (Figure S1), (ii) are in strong LD, and (iii) are physically linked in the genetic map of Harrang et al. (2015). These observations suggest the presence of chromosomal rearrangements (Ma & Amos, 2012; Mérot, 2020; Nowling et al., 2019). With the former population genetic studies using far less markers, those chromosome‐wide island of differentiation have been easily missed. It is however interesting to notice that one mitochondrial marker (12S rRNA) allowed to detect this parallelism (Diaz‐Almela et al., 2004). Such patterns have been already observed in fishes (Berg et al., 2017; Duranton et al., 2018; Hemmer‐Hansen et al., 2013; Jones et al., 2012; Riquet et al., 2019), often involving chromosomal rearrangements. Linkage and recombination suppression between markers allow allelic combinations to be maintained in face of gene flow. Increasing examples show off that chromosome rearrangements such as inversions have a pervasive role in eco‐evolutionary processes (Wellenreuther & Bernatchez, 2018). Interestingly, mapping of the linked markers onto the genome of *C. gigas* shows that they are also physically linked in that species (markers from the same LGD in *O. edulis* are found on the same LG in *C. gigas* as well), so that synteny of those regions seems to have been conserved between the two species, although they do not belong to the same genus, putatively indicating the importance of the structural integrity of this region. Our study proposes hypotheses that need to be tested and deepened as we performed a preliminary indirect approach (Mérot et al., 2020).

### 4.3 Management of restoration programs and research needs

Although more work is needed to understand the origin and the factors that maintain the heterogeneous and contrasted patterns of genetic differentiation between the four genetic clusters, the identified mosaic of those clusters is genuine and they can be considered as Evolutionary Significant Units (ESUs). They should accordingly be managed with dedicated conservation strategies. Therefore, there is a need to create and conduct monitoring protocols as soon as the very beginning of a program (Bernatchez et al., 2019). This should first include a genetic characterization of the oysters released (genetic diversity and cluster membership) in order to avoid potential maladaptation when transplantation and/or a too low diversity that could impair the sustainability of the program. When considering the potential low recruitment observed in northern Europe and the sweepstake reproductive success in this species (Hedgecock & Pudovkin, 2011), a temporal genetic monitoring of the spat recruited should also be conducted in order to follow the evolution of the genetic diversity. This is particularly important for the numerous programs now settling in England, The Netherlands, Ireland, Germany, Scotland, Sweden, Belgium, or France (16 programs counted in 2022 on the NORA website; https://noraeurope.eu/). Being able to detect human translocations due to aquaculture (such as the one we identified in Italy) might also be very useful in restoration programs.

Therefore, raising awareness of genetic considerations among managers of restoration programs should be continued, together with the transfer of easy to use and relatively cheap genetic molecular tools. Besides, a better characterization of the boundaries between the different genetic units in the European flat oyster needs to be conducted. This is particularly true in the English Channel and on the Atlantic coasts of Spain and Portugal where a sampling effort needs to be performed but is already somehow circumscribed. This is clearly more challenging in the Mediterranean and Black Seas where much more coasts still need to be covered. A more prospective issue could be the choice of the origin of the oysters for restocking in case one ESU becomes critically endangered. Should we choose the genetically or geographically closest population or should we take the one that potentially share barrier loci? For example, the Black Sea populations of flat oysters are declining dramatically (Todorova et al., 2009), should a restocking program with non‐native oysters prefer West Med oysters or should we choose North Sea oysters instead, or maybe purposely admix genomes of the two? Finally, as shellfish restoration programs tend to be supported by mass release of hatchery‐produced juveniles, there is an urgent need for more theoretical and applied research to evaluate genetic impacts of such practices.

## CONFLICT OF INTEREST

The authors do not declare any conflict of interest.

## Supporting information


Figure S1

Figure S2

Figure S3

Figure S4

Table S1
Table S2
Table S3
Table S4
Table S5

## Data Availability

Data for this study are available at https://www.seanoe.org/data/00737/84947/.
