# Supplementary figures and images for: Genetic parallelism between European flat oyster populations at the edge of their natural range
Source: Evol Appl. 2022 Aug 6;16(2):393–407. doi: 10.1111/eva.13449 (PMC9923475; doi:10.1111/eva.13449)

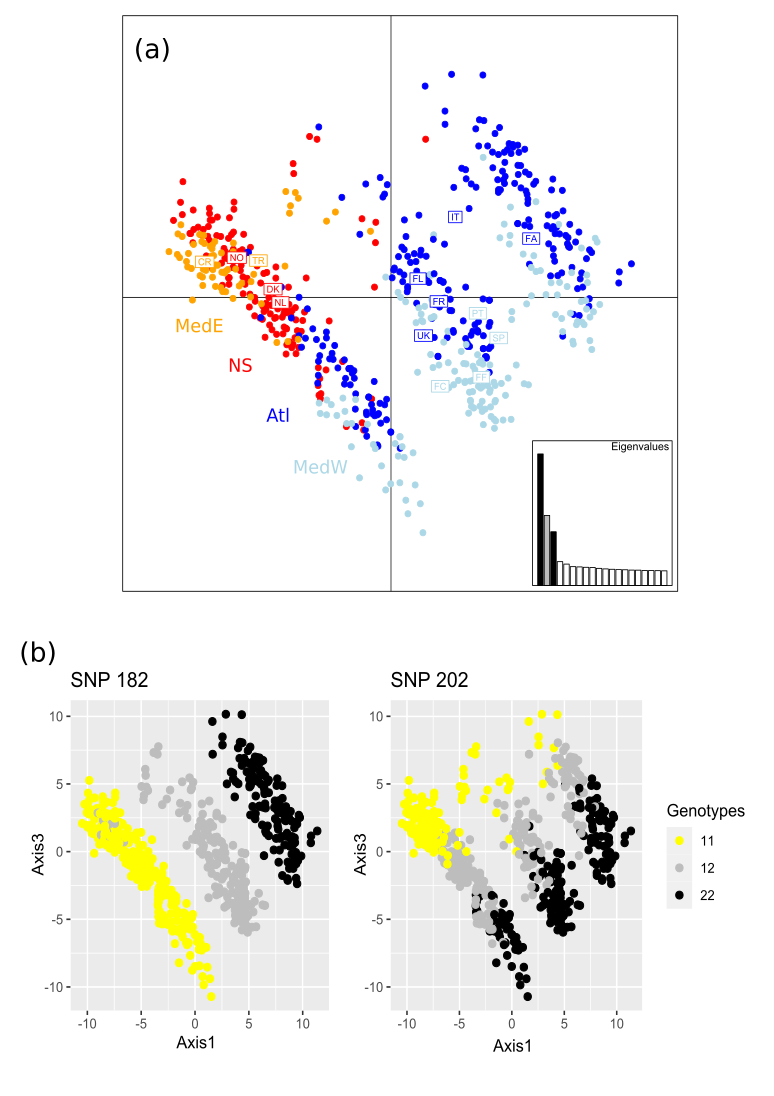

Supplement: Supplementary file 1 — Figure S1 [file EVA-16-393-s001.png]

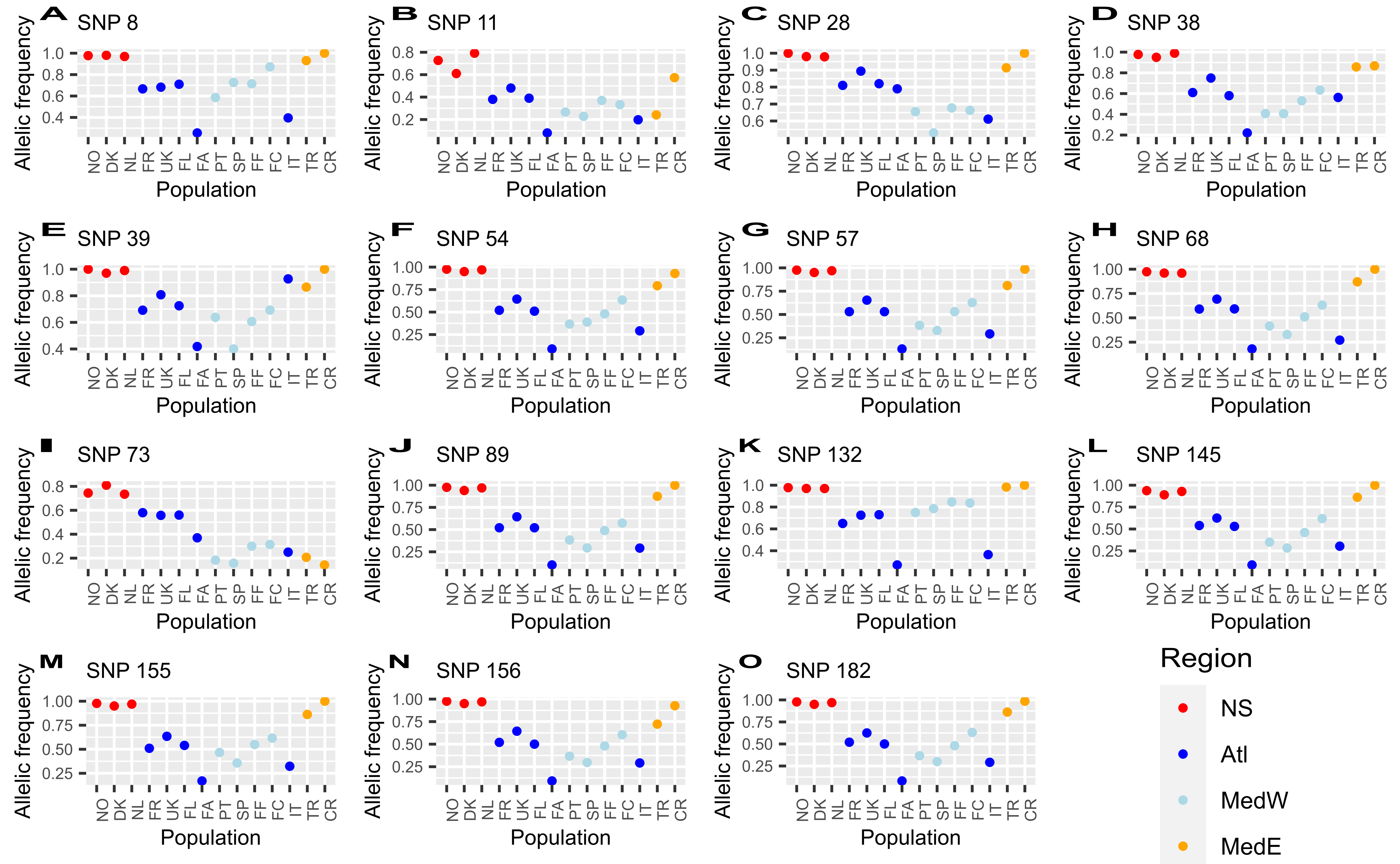

Supplement: Supplementary file 2 — Figure S2 [file EVA-16-393-s005.png]

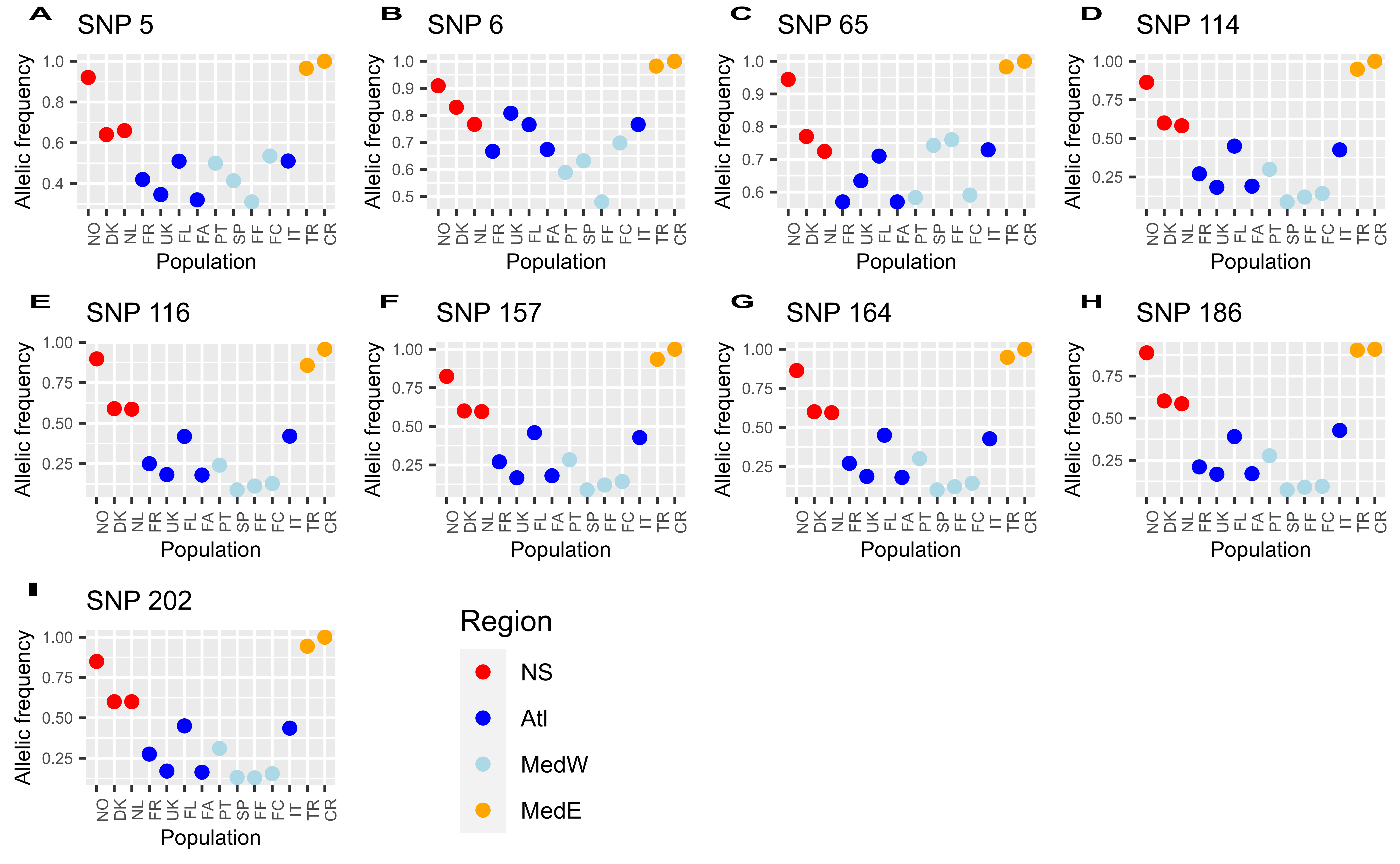

Supplement: Supplementary file 3 — Figure S3 [file EVA-16-393-s004.png]

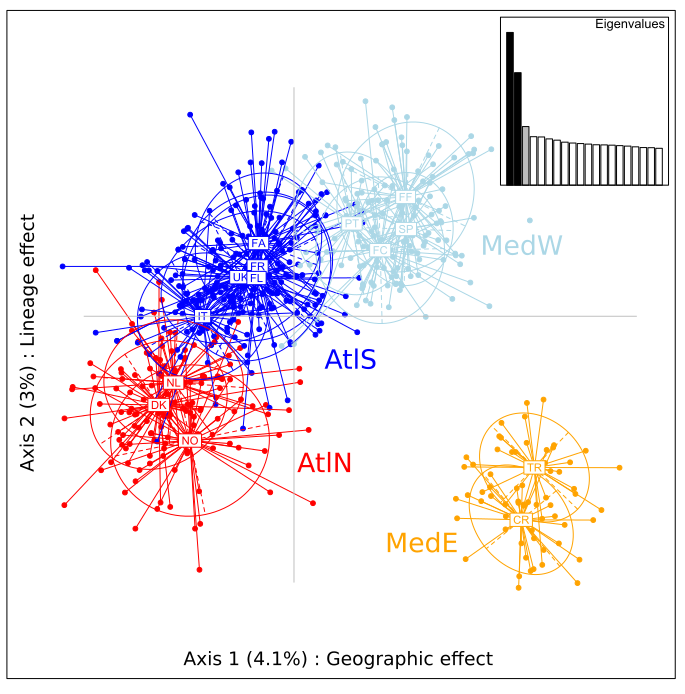

Supplement: Supplementary file 4 — Figure S4 [file EVA-16-393-s002.png]
